# Once upon a Testis: The Tale of Cyclic Nucleotide Phosphodiesterase in Testicular Cancers

**DOI:** 10.3390/ijms24087617

**Published:** 2023-04-20

**Authors:** Federica Campolo, Maria Rita Assenza, Mary Anna Venneri, Federica Barbagallo

**Affiliations:** 1 Department of Experimental Medicine, Sapienza University of Rome, 00161 Rome, Italy; federica.campolo@uniroma1.it (F.C.); maryanna.venneri@uniroma1.it (M.A.V.); 2 Faculty of Medicine and Surgery, “Kore” University of Enna, 94100 Enna, Italy; mariarita.assenza@unikore.it

**Keywords:** phosphodiesterases (PDEs), cAMP, cGMP, testis, testicular cancer

## Abstract

Phosphodiesterases are key regulators that fine tune the intracellular levels of cyclic nucleotides, given their ability to hydrolyze cAMP and cGMP. They are critical regulators of cAMP/cGMP-mediated signaling pathways, modulating their downstream biological effects such as gene expression, cell proliferation, cell-cycle regulation but also inflammation and metabolic function. Recently, mutations in PDE genes have been identified and linked to human genetic diseases and PDEs have been demonstrated to play a potential role in predisposition to several tumors, especially in cAMP-sensitive tissues. This review summarizes the current knowledge and most relevant findings regarding the expression and regulation of PDE families in the testis focusing on PDEs role in testicular cancer development.

## 1. Introduction

Cyclic adenosine monophosphate (cAMP) and cyclic guanosine monophosphate (cGMP) are intracellular second messengers that play a central role in a plethora of signaling pathways, involved in cell proliferation and differentiation, cell-cycle regulation, Ca^2+^-dependent signaling, inflammation and metabolic function [1,2,3]. Intracellular levels of cAMP and cGMP are finely regulated by adenylyl (AC) and guanylyl-cyclases, which catalyze the synthesis of cAMP and cGMP from ATP and GTP, respectively. The increase in intracellular cAMP and cGMP levels triggers the activation of several cellular effectors, among which the mains are cAMP- and cGMP-activated protein kinases, PKA and PKG, respectively [4,5]. The maintenance of cyclic nucleotide levels in physiological ranges is dependent on the activity of phosphodiesterases (PDEs) that catalyze the hydrolysis of cyclic nucleotides to the corresponding inactive non-cyclized monophosphate form (i.e., 3′,5′-cGMP to 5′-GMP and 3′,5′-cAMP to 5′AMP, respectively) [6,7]. Mammalian PDEs are classified into 11 families encoded by 21 different genes, that are grouped based on their amino acid sequences, biochemical properties, affinities for cAMP and cGMP and response to specific activators, inhibitors and effectors. Each PDE family consists of multiple isoforms generated by alternative mRNA splicing or transcriptional processing, giving rise to over 100 isoenzymes, which display different tissue expressions and intracellular localization [7,8]. PDEs share a common structural organization, with a conserved carboxy-terminal catalytic domain, while amino-terminal hydrophobic regulatory regions contain structural determinants that target individual PDEs to different subcellular locations allowing individual PDEs to specifically respond to different post-translational modifications and signaling molecules [9,10,11]. Many of the PDE families contain amino-terminal subdomains, such as GAF domains, which regulate the allosteric binding of cGMP to PDE2, PDE5, PDE6 and PDE11, or of cAMP to PDE10 [12]; upstream conserved regions, (e.g., in PDE4), harbors a PKA consensus site [13]; Per-Arnt-Sim domains and receiver domains (e.g., in PDE8) [14]. Moreover, some PDE families contain phosphorylation sites, able to increase their enzyme activity, such as PDE5 phosphorylation site (Ser92) to PKG [15]; PDE1 presents two Calmodulin (CaM)-binding domains activated by changes in intracellular [Ca^2+^] [16]; in PDE4, the presence of extracellular signal-regulated kinase 2 plays a role in regulating their activity and subcellular targeting [17]. The regulation of cyclic nucleotide signaling is thought to be one of several pathways involved in tumor cells dissemination and function. In recent years, we have witnessed a growing interest in the use of pharmacological inhibition of PDEi as an anticancer strategy in several tumors [18].

In this review we first focused on the main findings of PDEs expression and the role in the testis; then, we summarized the evidence of the aberrant expression of this class of enzymes in testicular cancer.

Throughout this review, PDEs mRNA are capitalized and italicized, while the protein products are capitalized but not italicized.

## 2. An Overview of Phosphodiesterase Families in Testis

### 2.1. PDE1

**Figure 1:**
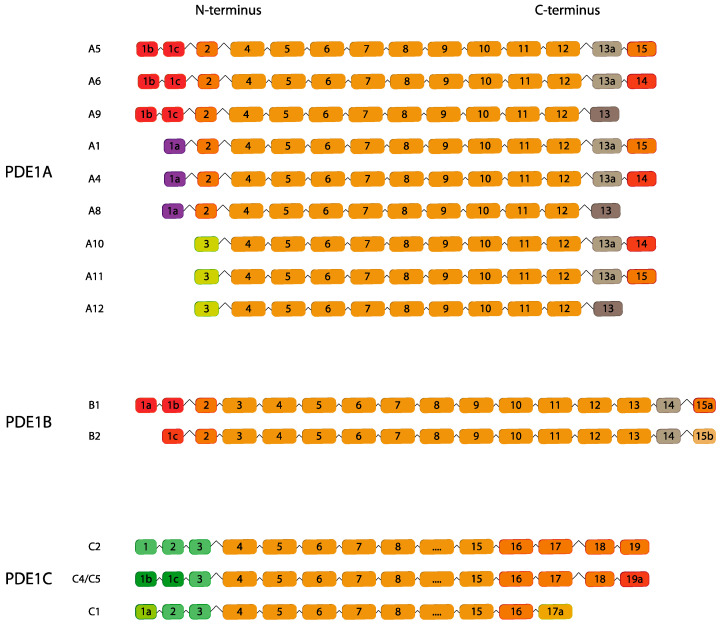
Ca^2+^/CaM-stimulated *PDE1* family. Human variants are depicted except for *PDE1C* (mouse variants). This figure was gathered through analysis and cross-referencing of online databases (https://www.ensembl.org/ and https://www.uniprot.org, accessed on 30 March 2023) for *PDE1B* and *PDE1C*; *PDE1A* was adapted from [21]. Boxes and lines represent exons and introns, respectively. The boxes with different colors indicate alternative exons. The maximum number of exons illustrated is 17 and ellipsis indicate exons not shown.

PDE1 is one of the first families to be identified and comprises Ca^2+^ and calmodulin-regulated PDEs displaying Ca^2+^/CaM binding domains. This family consists of three subfamilies encoded by three different genes: *PDE1A*, *PDE1B*, and *PDE1C* (Figure 1). Each PDE1 isoenzyme is present in tissues as splice variants that differ in molecular weight, cellular and subcellular distribution and may play different roles accordingly. PDE1 enzymes are able to hydrolyze both cAMP and cGMP, with different affinities: PDE1A and PDE1B exhibit higher affinity for cGMP whereas PDE1C possesses a similar affinity for cAMP and cGMP.

Thus far, nine human and four mouse splice variants encoding for PDE1A have been annotated, while two human and three mouse *PDE1B1* splice variants are known [19]. Four PDE1C splice variants have been reported in mouse, named *PDE1C1–5* [18], while in human, 10 potential isoforms rising from an alternate promoter or alternate transcription start site have been suggested; however, a consensus on their nomenclature is still missing [20,21]. PDE1A is widely expressed in various tissues, with some specific isoform restricted to the brain [21]. Although several differences between mouse and human variants have been highlighted it is commonly recognized that *PDE1A10* (*PDE1A9* in mice) is testis-specific and its localization and function are conserved between the two species [22] (Table 1 and Table 2). The elective tissue for PDE1B expression is the brain both in humans and mice with low or undetectable levels in the testis regardless of the splice variants [23,24]. In humans, PDE1C expression is high in both the heart and brain, while murine PDE1C1 and PDE1C5 isoforms have been detected at high levels in the cerebellum [25].

The first evidence for the presence of calmodulin-dependent phosphodiesterases within the testis emerged from biochemical studies by Purvis and collaborators in the 1980s [26,27]. They were able to isolate, by diethylaminoethyl cellulose chromatography, three Ca^2+^-CaM dependent isoenzymes in immature rat testis, later on only the high-affinity cGMP isoform was confirmed in both somatic and germ cell-enriched populations isolated from 12-day-old rat testis [28,29] (Table 1). Some years later, the same group better clarified that somatic cells possess a Ca^2+^-CaM-dependent high-affinity cGMP phosphodiesterase whereas germ cells present a Ca^2+^-CaM-dependent high affinity for cGMP and a high and low affinity for cAMP [30]. Despite the slight difference in the results, the two groups agreed on the fact that CaM-PDE activities are developmentally regulated in rodent germ cells.

**Table 1:** Expression of PDE family in rodent testis.

Gene Family	Gene	Cell Type	References
PDE1	Un	GCs	[28]
	PDE1A	rSPT, eSPT, mSPT, SPZ	[22,31,32]
	PDE1C	SPC, rSPT, eSPT, mSPT	[32]
PDE2	PDE2A	SPZ, Un	[31,33]
PDE3	Un	SPZ	[31]
	PDE3A	SPC, Un	[34]
	PDE3B	SPC	[34]
PDE4	Un	Un, SPZ	[35,36]
	PDE4A	rSPT, pSPC	[37,38,39,40]
	PDE4B	SCs, LCs, SPZ	[31,35,41]
	PDE4C	pSPC, SPT, LCs, SPZ	[31,38,39,41]
	PDE4D	pSPCs, rSPT, eSPT, mSPT, SPZ, SCs	[31,40,42,43,44,45]
PDE5	PDE5A	LCs, peritubular cells, Un	[46,47,48,49]
PDE6	PDE6A	SPZ, Un	[31]
	PDE6C	LCs, SPZ	[31,49]
	PDE6D	LCs, SPZ, Un	[31,49]
	PDE6G	Un	[31]
	PDE6H	Un	[31]
PDE7	Un	Un	[50]
	PDE7B	pSPC	[51]
PDE8	PDE8A	LCs, pSPC, SPZ	[31,41,49,52,53]
	PDE8B	Un, LCs SPZ	[31,41,49]
PDE9	PDE9A	Un, LCs	[31,49]
PDE10	PDE10A	SPZ, Un, LCs	[31,49,54,55,56]
PDE11	PDE11A	SPZ	[31,57]

SPC: spermatocytes; pSPC: pachytene spermatocytes; SPT: spermatids; rSPT: round spermatids; eSPT: elongated spermatids; SPZ: spermatozoa; GCs: germ cells; LCs: Leydig cells; SCs: Sertoli cells; UN: unspecified.

We need to wait 15 years until all PDE1 isoenzymes are cloned [58,59,60,61,62,63] before having a better characterization of the stage and cell-specific expression of PDE1 enzymes in murine testis [32]. A spatial and temporal expression pattern was observed for *PDE1A* and *PDE1C*, with *PDE1B* considered absent. In particular, *PDE1A* mRNA was found in round-to-elongated spermatids, while the protein expression was detected in the tails of elongated and maturing spermatids but not in spermatocytes and spermatogonia [22,32]. *PDE1C* was expressed in the early-meiotic prophase through the meiotic and post-meiotic stages [32].

In addition, a particulate CaM-PDE activity was noticed in the head and tailpieces of rat caudal epididymal sperm [15]. These observations suggest that CaM-PDEs likely have important roles in spermatogenesis and in the maturation of spermatozoa. Additionally, capacitation was partially mediated by CaM-PDE activities [15].

In human spermatozoa PDE1 inhibitors selectively stimulated the acrosome reaction, and given that PDE1A is the major form expressed in mature sperm; this variant was attentional as an ideal candidate to play an important role in cyclic nucleotide regulation of mature sperm function [22,36] (Table 2).

**Table 2:** Expression of PDE family in human testis.

Gene Family	Gene	Cell Type	References
PDE1	PDE1A	SPZ	[36]
PDE2	PDE2A	Un	[64,65]
PDE3	PDE3A	SPZ	[66,67]
PDE4	PDE4A	Un, SPZ	[64,65,66]
	PDE4B	Un, SPZ	[65,66]
	PDE4C	Un, SPZ	[64,66]
	PDE4D	Un	[65]
PDE5	PDE5A	SPZ	[66]
PDE6	PDE6B	Un	[64]
PDE7	PDE7B	Un	[64]
PDE8	PDE8A	Un, LCs	[64,65,68]
	PDE8B	Un, LCs	[65]
PDE9	PDE9A	Un	[20,64]
PDE10	PDE10A	Un	[64]
PDE11	PDE11A	SPC, SPT, LCs, Un	[64,65,69]

SPC: spermatocytes; SPT: spermatids; SPZ: spermatozoa; LCs: Leydig cells; UN: unspecified.

### 2.2. PDE2

PDE2 hydrolyzes both cAMP and cGMP with higher affinity for the latest. It belongs to the so-called “cGMP-stimulated PDE” since the binding of cGMP to the allosteric GAF-B domain causes a conformational change that in turn stimulates cAMP hydrolysis. Given this unique feature, PDE2A serves as a key regulator for the cAMP-cGMP crosstalk [70].

**Figure 2:**
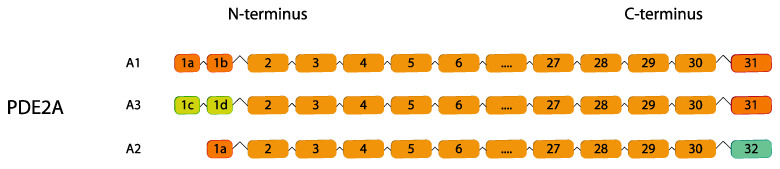
cGMP-stimulated *PDE2* family. Human variants are depicted. This figure was gathered through analysis and cross-referencing of online databases (https://www.ensembl.org/, and https://www.uniprot.org, accessed on 30 March 2023). Boxes and lines represent exons and introns, respectively. The boxes with different colors indicate alternative exons. The maximum number of exons illustrated is 17 and ellipsis indicate exons not shown.

Three isoforms of PDE2 have been isolated so far: *PDE2A1*, *PDE2A2* and *PDE2A3* (Figure 2). These isoforms differ in their N-terminus which mediates their subcellular localization [71]. *PDE2A* mRNA expression is similar in human and rodent tissue, including the heart, liver, adrenal gland, platelets, brain, endothelial cells, neurons and macrophages [70,72,73,74,75,76].

Few reports characterized PDE2A expression in testis. Microarray data regarding the expression of PDE genes in different human tissues reported a high level of *PDE2A* in the human testis [64], later confirmed by the QuantiGene Bioplex Assay [65] (Table 2). Positive results were obtained in human ejaculated spermatozoa [66] while low expression levels were detected in murine spermatozoa [31] (Table 1). By Western blot an, undetectable signal was found in mouse testis extract, but there was mild to moderate staining by immunohistochemistry on rat, mouse and human testis slices (from lower to highest). In particular, authors reported positive staining in subsets of spermatogenic and in Sertoli cells, albeit this statement was not properly supported by images making it difficult to discriminate to which cells the authors are referring to [33]. Even if its expression was confirmed, the role of PDE2 in testis is still unknown.

### 2.3. PDE3

PDE3 hydrolyzes both cyclic nucleotides with higher affinity with cAMP. It has earned the denomination of “cGMP-inhibited PDE” due to a distinctive feature: binding of cGMP is able to inhibit cAMP hydrolysis.

**Figure 3:**
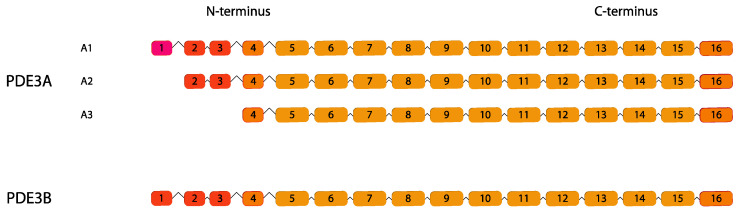
cGMP-inhibited PDE gene family. Human variants are depicted. This figure was gathered through analysis and cross-referencing of online databases (https://www.ensembl.org/ and https://www.uniprot.org, accessed on 30 March 2023). Boxes and lines represent exons and introns, respectively. The boxes with different colors indicate alternative exons. The maximum number of exons illustrated is 17 and ellipsis indicate exons not shown.

Enzymes belonging to the PDE3 family are transcribed from two different genes, *PDE3A* and *PDE3B* [77,78]. For PDE3A, three variants have been described, differing only in the lengths of their N-terminal sequences [10,79] and not in their basal catalytic activity [80]. Up to now, PDE3B is the only isoform annotated [77] and possesses similar catalytic activity to PDE3A (Figure 3) [81]. PDE3A is expressed in the heart, vascular smooth muscle regulating myocardial and smooth muscle contractility, platelets, oocyte and kidney whereas PDE3B is enriched in vascular smooth muscle, adipocytes, hepatocytes, kidney, b cells, T lymphocytes and macrophages and it is involved in hormonal regulation of lipolysis and glycogenolysis [82,83].

The first evidence of PDE3 transcripts in testis arises from the results obtained on rat testis where *PDE3A* was detected in vessels and *PDE3B* was found in primary spermatocytes [34] (Table 1). No other characterization of the cellular localization of these two enzymes was carried out, leaving it unclear if the transcripts are also translated into functional proteins in these subsets of cells. Enzyme activity, immunocytochemical localization and immunoblotting for PDE3A were applied on human spermatozoa raveling that it is expressed and localized on the post-acrosomal segment of the sperm head [66,67] (Table 2); however, its inhibition by milrinone did not significantly stimulate capacitation or hyperactivation suggesting that PDE3 does not have a major role in sperm function [67].

### 2.4. PDE4

**Figure 4:**
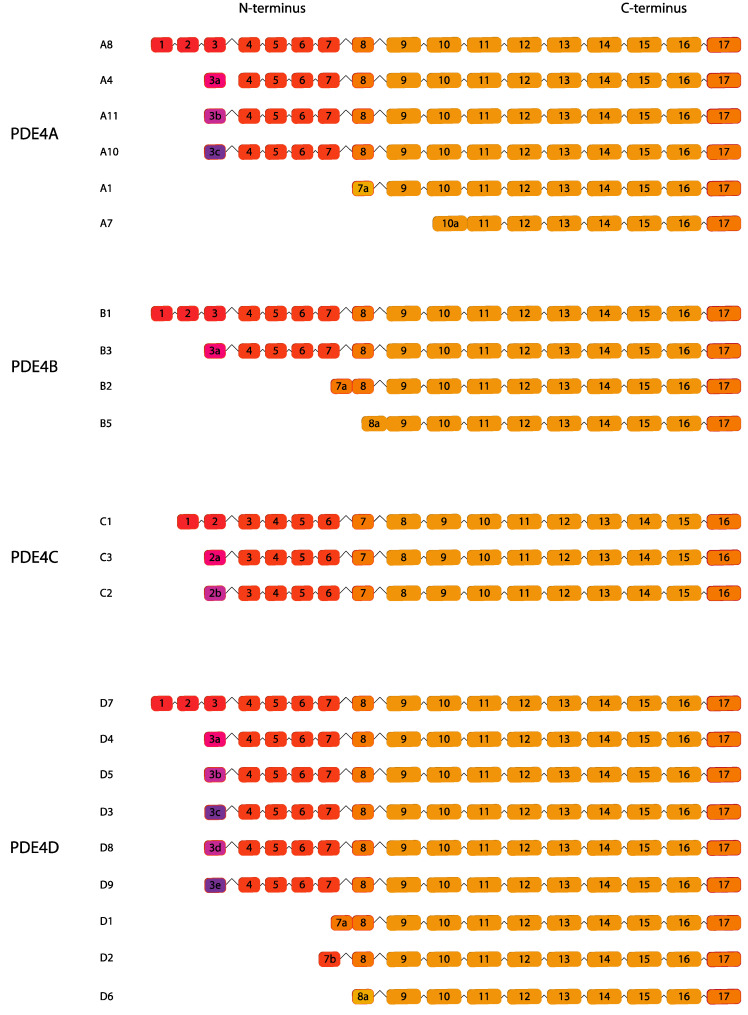
PDE4 gene family. Human variants are depicted. This figure was gathered through analysis and cross-referencing of online databases (https://www.ensembl.org/ and https://www.uniprot.org, accessed on 30 March 2023). Boxes and lines represent exons and introns, respectively. The boxes with different colors indicate alternative exons. The maximum number of exons illustrated is 17 and ellipsis indicate exons not shown (adapted from [84]).

PDE4 enzymes constitute the majority of cAMP-selective PDEs. Four distinct genes (*PDE4A*, *PDE4B*, *PDE4C* and *PDE4D*) encode the PDE4 family of enzymes, each of these genes produce a plethora of transcript variants and different protein isoforms (Figure 4). PDE4 subtypes and isoforms possess tissue- and cell type-specific expression but also they can have intracellular compartmentalization specificity. PDE4 enzyme expression is ubiquitous with variant-specific tissue distribution [82].

In 1992, Conti’s group performed a Northen blot assay, using a probe specific for PDE4A, to analyze its expression on isolated spermatogenic cells. They detected a 4.0-kb *PDE4* mRNA in mouse and rat pachytene spermatocytes and five transcripts mRNAs in round spermatids, while a lower amount of transcripts was found in condensing spermatocytes/residual bodies [37] (Table 1). Similar results were obtained by an in situ hybridization approach performed by Morena and colleagues. They detected a high *PDE4A* signal in round spermatids, later attributed to *PDE4A7* isoform [38], that declined in elongating spermatids [39,40]. Western blotting using PDE4 subtype-selective antibodies confirmed the pattern of mRNA expression studies conducted by in situ hybridization [35]. Developmental studies on rodents are also consistent with the presence of *PDE4A8* mRNA and expression of PDE4A8 and 88-kDa PDE4A protein at 20–30 days of age. *PDE4B* mRNAs and protein were found primarily in the Sertoli and Leydig cells [35], whereas *PDE4C* maximal expression was detected in stages VIII-XIII of the seminiferous epithelium indicating that it is expressed preferentially in middle–late pachytene spermatocytes [39]. As for PDE4B also PDE4D, in particular PDE4D1 and PDE4D2, are expressed in immature Sertoli cells and regulated by a follicle-stimulating hormone (FSH)-cAMP-mediated mechanism [42,43,44]. Given the different electrophoretic mobilities, five immunoreactive species were detected in immature Sertoli cells [45].

The longest *PDE4D* transcripts were also enriched in pachytene spermatocyte and round spermatids [40]. Translated protein appears only in a region surrounding the acrosome of elongating and maturing spermatids in close proximity to microtubules present in the transitory structure of the manchette indicating that *PDE4D* mRNA is not efficiently translated at round spermatids stages but it reaches a maximal intensity at steps 18–19 before spermiation [40]. *PDE4A, PDE4B* and *PDE4D* have been detected with a similar rate of expression in whole human testis extract [65] (Table 2).

In mouse spermatozoa, immunolocalization of PDE4 revealed an intense signal for isoform D in the principal piece and across the entire acrosome and for 4A in the flagellum, while 4B and 4C were considered expressed at very low levels or absent [31]. *PDE4A*, *4B*, *4C* but not *4D* transcripts were detected in human spermatozoa [66] and their inhibition enhanced sperm motility, phosphorylation of membrane proteins [85] without affecting acrosome reaction [36]. Surprisingly, a proteomic study revealed only PDE4D but no other PDE4 isoforms expression in human sperm [86].

MA10 mouse Leydig cell line and primary rodent Leydig cells expressing PDE4B and 4C and PDE4 inhibition by rolipram are able to regulate steroid synthesis under basal conditions and upon luteinizing hormone (LH) stimulation [41]. PDE4 and PDE8A (see forehead), coordinate steroidogenesis through several layers of control such as transcription, lipid and glucose metabolism, endocytosis and vesicle transport to facilitate maximal steroid output and to assure timely and adequate testosterone secretion in response to LH [87].

### 2.5. PDE5

**Figure 5:**
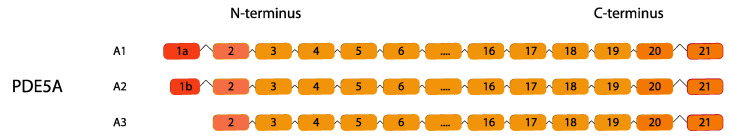
*PDE5* gene family. Human variants are depicted. This figure was gathered through analysis and cross-referencing of online databases (https://www.ensembl.org/ and https://www.uniprot.org, accessed on 30 March 2023). Boxes and lines represent exons and introns, respectively. The boxes with different colors indicate alternative exons. The maximum number of exons illustrated is 17 and ellipsis indicate exons not shown (adapted from [93]).

PDE5A is characterized by high specificity for cGMP [88,89]. Three murine and human isoforms have been characterized so far that are transcribed from different promoters, producing three N-terminal variants (*PDE5A1*, *PDE5A2*, *PDE5A3*) [47,90,91] (Figure 5). *PDE5A* transcripts are widely expressed but high levels have been detected in several sections of the digestive system, lung, platelets, cerebellum, kidney, vascular smooth muscle cells, skeletal and cardiac muscle [92,93,94,95,96,97,98], and several endocrine glands, including testis [47,65,99].

Immunolocalization of PDE5A in prepuberal and adult testis is restricted to Leydig and peritubular cells, proposing cGMP-mediated processes to influence not only the vessel dilatation, but also the testosterone synthesis by Leydig cells [46].

This idea was later confirmed in mice chronically treated with sildenafil. The treatment induced several changes in Leydig cells such as vesicular smooth endoplasmic reticulum, large vacuoles scattered through the cytoplasm and enlarged mitochondria, hallmarks of an activated steroid-secreting cell [48]. The fact that sildenafil-treated mice presented also increased levels of total testosterone suggest that PDE5/PKG could be involved in the modulation of androgen biosynthesis [48,49,100]. The effect of PDE5i on the human chorionic gonadotropin/LH-induced steroidogenic pathway was also investigated in HEK293 and MLTC-1 cell lines by Forster resonance energy transfer-based biosensors revealing that PDE5i was able to enhance the conversion of progesterone-to-testosterone in a cAMP-independent manner [101]. This effect was later explained by a cross-interaction between PDE5i and cAMP-specific PDE8A/PDE8B leading to an increase in cAMP and sex hormones levels [101,102]. Interestingly, recent results indicate that long-term sildenafil treatment improves testicular steroidogenesis as well as the sensitivity of Leydig cells to gonadotropic stimulation and ameliorate the atrophy of seminiferous tubules during aging [103].

Although Sertoli cells seem not to express PDE5A [46], some studies demonstrated that PDE5A regulates Sertoli cell secretion. In Azoospermic men, vardenafil modulates Sertoli cell secretory function and results in androgen-binding protein enhancement, a biological marker of Sertoli cell secretion [104] may be due an indirect positive effect on peritubular cells or on the secretory function of Leydig cells. The literature regarding PDE5A localization in human testis is lacking.

Given the high specificity and safety of PDE5 inhibitors, several clinical trials have been conducted. In two clinical trials performed on healthy volunteers, sildenafil does not modify seminal parameters and acrosome reaction [105,106]; on the other hand, either sildenafil citrate or 8-Bromo-cGMP treatments increased sperm-zona pellucida binding, suggesting that PDE5i can be used to enhance sperm motility and oocyte binding [106]. Jannini et al. investigated the effect of orally administered sildenafil in healthy men. In this study, no effect of sildenafil administration was observed regarding sperm motility, concentration, or in the total number of ejaculated spermatozoa. However, when sildenafil was administrated before the second postcoital test, it increase sperm number and motility [107]. Several attempts to clarify the role of PDE5A by inhibiting its activity have been performed in vitro on human spermatozoa, where its expression was confirmed by Richter and colleagues [66] (Table 2). The effects of sildenafil, vardenafil and tadalafil on the motility, viability, membrane integrity and functional capacity of human spermatozoa are controversial [108,109,110,111]. Some authors reported positive effects of sildenafil on sperm viability, sperm motility, sperm forward progression and acrosome reaction attributing these effects to cGMP-regulated calcium influx [112]. Others obtained no significant effects leaving the debate still open.

### 2.6. PDE6

**Figure 6:**
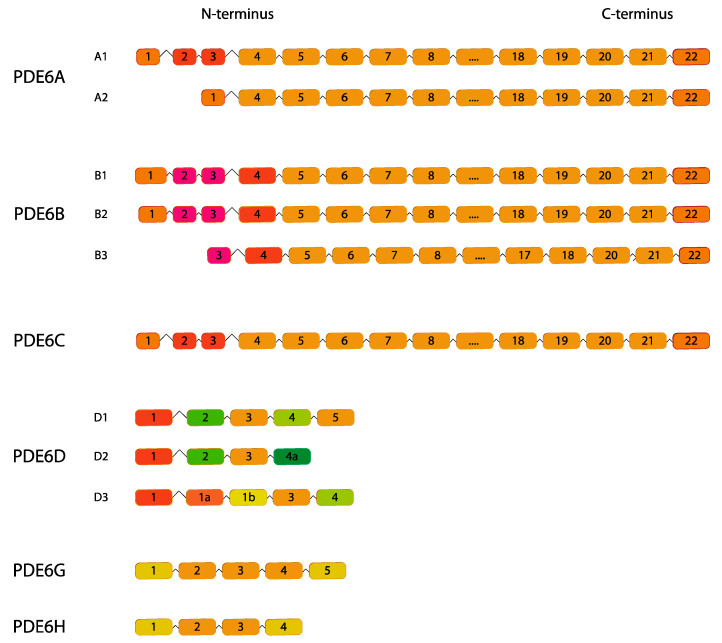
*PDE6* gene family. Human variants are depicted. This figure was gathered through analysis and cross-referencing of online databases (https://www.ensembl.org/ and https://www.uniprot.org, accessed on 30 March 2023). Boxes and lines represent exons and introns, respectively. The boxes with different colors indicate alternative exons. The maximum number of exons illustrated is 17 and ellipsis indicate exons not shown.

Photoreceptor cell-specific PDE6 protein complex comprises three genes: *PDE6A*, *PDE6B* and *PDE6C* encoding the catalytic subunits; *PDE6G* and *PDE6H* genes encoding the inhibitory subunits and *PDE6D* responsible for their solubilization (Figure 6) [113].

PDE6 is the primary regulator of cytoplasmic cGMP concentration in rod and cone photoreceptors being almost exclusively expressed in the mammalian retina and in the pineal gland [114]. *PDE6* expression in rodent testis has been first demonstrated by Andric et al. They found that chronic treatment with sildenafil of rat Leydig cells reduces *PDE6*C without affecting *PDE6D* mRNA expression [49] (Table 1). Moreover, Baxendale and Fraser investigated the presence and function of PDEs in mouse testis and mature spermatozoa demonstrating that *PDE6A* and *PDE6D* transcripts are expressed in both, *PDE6G* and *PDE6H* are expressed in testis only while *PDE6C* expression is restricted to mature spermatozoa [31].

Microarray analysis of phosphodiesterases expression on human tissues revealed that *PDE6A* and *PDE6C* are undetectable while *PDE6B* is expressed at moderate levels in normal testis [64] (Table 2).

### 2.7. PDE7

**Figure 7:**
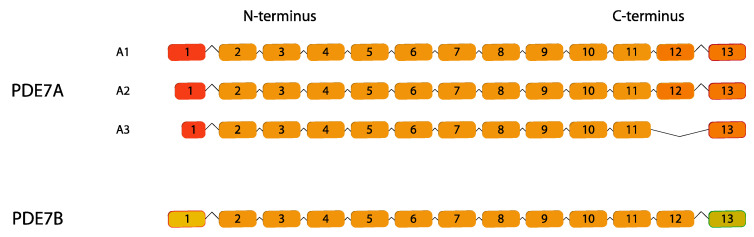
*PDE7* gene family. Human variants are depicted. This figure was gathered through analysis and cross-referencing of online databases (https://www.ensembl.org/ and https://www.uniprot.org, accessed on 30 March 2023). Boxes and lines represent exons and introns, respectively. The boxes with different colors indicate alternative exons. The maximum number of exons illustrated is 17 and ellipsis indicate exons not shown.

PDE7 is a cAMP-selective phosphodiesterase encoded by two genes *PDE7A* and *PDE7B* that, following alternative splicing, give rise to three *PDE7A* isoforms (*PDE7A1*, *PDE7A2*, and *PDE7A3*); for *PDE7B* it is widely recognized only one translated isoform [115] (Figure 7).

Timothy Bloom and Joseph Beavo first reported a barely detectable *PDE7* expression signal in mouse testis performing a ribonuclease protection analysis [50] (Table 1), also revealing high expression levels of *PDE7* in mouse skeletal muscle. In human tissues, the highest expression levels are detectable in T lymphocytes [116]. Some years later, Sasaki and coworkers characterized *PDE7* expression in rat testis. Using northern and in situ hybridization analyses, they showed that *PDE7B* transcripts were particularly abundant in rat spermatocytes [51]. The expression of PDE7 in the human testis is still debated. The only data come from a microarray analysis of PDE expression on human tissues that showed that *PDE7B* is expressed at moderate levels in normal testis while *PDE7A* levels are undetectable [64] (Table 2).

### 2.8. PDE8

**Figure 8:**
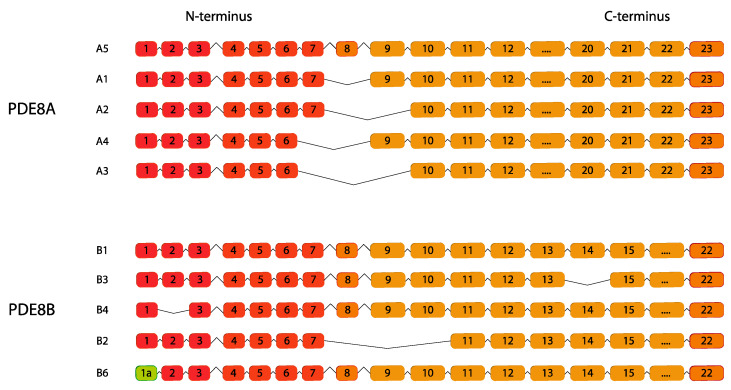
*PDE8* gene family. Human variants are depicted. This figure was gathered through analysis and cross-referencing of online databases (https://www.ensembl.org/ and https://www.uniprot.org, accessed on 30 March 2023). Boxes and lines represent exons and introns, respectively. The boxes with different colors indicate alternative exons. The maximum number of exons illustrated is 17 and ellipsis indicate exons not shown (adapted from [67]).

PDE8 is a highly selective cAMP hydrolyzing enzyme and consists of two genes, *PDE8A* and *PDE8B* that due to alternative splicing processes, generate five splice variants: *PDE8A1–PDE8A5* and *PDE8B1–PDE8B5* [117,118] (Figure 8). Both *PDE8* genes are widely expressed in all steroidogenic cell types with PDE8A mostly expressed in the testis and T-cells and PDE8B mainly distributed in the brain and thyroid gland making PDE8 a key player in T-cell activation, thyroid hormones production, sperm and Leydig cell functions and cardiac functions [119,120].

The expression and the role of PDE8 in mouse testis were deeply analyzed by Vasta et al. that, using *PDE8A* knockout mice, provided evidence of PDE8A pivotal role in steroidogenesis [52] (Table 1). Some years later, Shimizu-Albergine et al. added important details to this issue demonstrating that PDE8A and PDE8B work in concert to regulate steroid production [41]. This was further supported by the finding that, although PDE8A and PDE8B hydrolyze distinct cAMP pools to regulate basal rates of steroidogenesis, maximal steroid production requires the inhibition of both isoforms [121,122]. The functional involvement of PDE8 in the regulation of mouse steroidogenesis was also supported by a phosphoproteomics analysis on the steroidogenic MA10 cell model stimulated with selective PDE8i. This analysis, tracing global phosphoproteome dynamics in response to cAMP/PKA activation, clearly demonstrated a specific role of PDE8 in the regulation of steroidogenic gene transcription [87].

Several studies investigated the role of PDE8 in the whole testis. In one of those studies, *PDE8A* mRNA was specifically found in pachytene spermatocytes, suggesting a potential role in germ cell development [53].

The expression of PDE8 has also been immunodetected in mouse spermatozoa by different groups [41]. To this regard, Baxendale and Fraser, investigating the presence and function of PDEs in mouse testis and mature spermatozoa, demonstrated that *PDE8A* and *PDE8B* transcripts are both expressed in mature testis while only *PDE8*A is expressed in mature spermatozoa [31]. *PDE8* expression in rodent testis has been further demonstrated by Andric et al. that analyze PDEs expression following chronic treatment with sildenafil demonstrating that rat Leydig cells express *PDE8*A and *PDE8B* mRNAs and sildenafil is not able to modify their levels [49].

Although there are many demonstrations on rodents, PDE8 expression in human testis has been poorly investigated due to the known limitations related to the availability of human specimens. A demonstration of the presence of PDE8 in human testis comes from studies conducted by Wang et al. that, analyzing the tissue distribution of human PDE8A isoforms, found that *PDE8A1* transcript is most abundant in testis while *PDE8A2* expression levels are highest in spleen followed by testis [68]. Moreover, a microarray analysis of phosphodiesterases expression on human tissues showed that *PDE8A* is expressed at moderate levels in normal testis while *PDE8B* levels are undetectable. We recently demonstrated that human Leydig cells express both PDE8A and PDE8B isoforms and that PDE8A is also highly expressed in specific spermatogenic stages, suggesting a potentially pivotal role of PDE8A in controlling key events of maturation of human sperm [65] (Table 2).

### 2.9. PDE9

**Figure 9:**
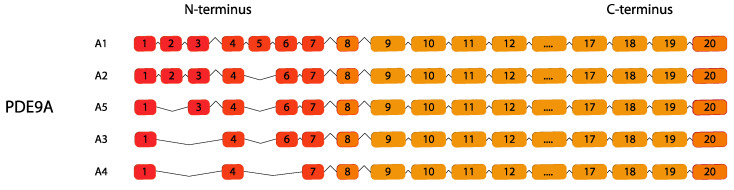
*PDE9* gene family. Human variants are depicted. This figure was gathered through analysis and cross-referencing of online databases (https://www.ensembl.org/ and https://www.uniprot.org, accessed on 30 March 2023). Boxes and lines represent exons and introns, respectively. The boxes with different colors indicate alternative exons. The maximum number of exons illustrated is 17 and ellipsis indicate exons not shown (adapted from [122]).

PDE9 is the cGMP-hydrolyzing PDE with the highest affinity for cGMP among all PDE families and is encoded by a *PDE9A* gene that, following alternative splicing, gives rise to five encoding transcripts (*PDE9A1–PDE9A6*, the latest originally named *PDE9A5*) [123] (Figure 9). Human PDE9A shows the highest expression levels in the spleen and brain particularly in Purkinje neurons and cerebellum [124,125].

PDE9A protein expression is highly conserved among species being widely distributed throughout mouse and rat brains with different regional expressions [126,127]. Studies on *PDE9A* knockout mice revealed that cGMP levels were increased in the brain cortex, hippocampus striatum, cerebellum and cerebrospinal fluid and the chronic treatment of wild-type mice with a PDE9 selective inhibitor (PF-4181366) increased cGMP levels in the same brain regions as well as in the cerebrospinal fluid [128]. The analysis of PDEs expression in rodent testis revealed that *PDE9A* mRNA is detectable in mouse testis [31] and in Leydig cells obtained from rats [49] while expression data on human testis are controversial [20,64] (Table 1 and Table 2).

### 2.10. PDE10

**Figure 10:**
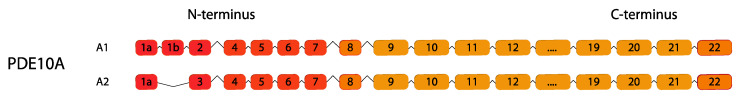
PDE10 gene family. Human variants are depicted. This figure was gathered through analysis and cross-referencing of online databases (https://www.ensembl.org/ and https://www.uniprot.org, accessed on 30 March 2023). Boxes and lines represent exons and introns, respectively. The boxes with different colors indicate alternative exons. The maximum number of exons illustrated is 17 and ellipsis indicate exons not shown (adapted from [130]).

PDE10 is a dual cAMP/cGMP hydrolyzing enzyme encoded by a single gene, *PDE10A* present in two major variants, *PDE10A1* and *PDE10A2* [54,55] (Figure 10). It shows a higher affinity for cAMP and may function in vivo as a cAMP-inhibited cGMP PDE [129].

PDE10A is mainly expressed in the thyroid, pituitary glands and brain; it has been suggested as a regulator of learning and memory processes [131].

The expression of *PDE10A* has been investigated by Baxendale and Fraser, analyzing the presence and function of murine PDEs and demonstrating that *PDE10A* transcripts are expressed in testis but not in mature spermatozoa [31] (Table 1). Other works confirmed this expression pattern in rodent testis with different technical approaches [54]. Among them, Andric et al. clearly demonstrated that rat Leydig cells express *PDE10* transcript and chronic treatment with a selective Pde5i is not able to modify *PDE10* mRNA expression levels [49]. A decade before, Fujishige et al. reported a strong PDE10A immunoblot signal corresponding to high enzymatic activity in rat testis and striatum [132], removing any reasonable doubt on the presence of PDE10 in these two organs.

PDE10A immunoreactivity is absent in the epididymal spermatozoa of mice; however, human spermatozoa have been demonstrated to express PDE10 [56]. Data on humans also come from a microarray analysis of PDEs expression showing that *PDE10A* is expressed at moderate levels in normal testis [64] (Table 2).

The exact function of PDE10 in spermatogenesis remains unclear; what is known from preclinical studies is that its constitutive deletion does not affect sperm’s ability to fertilize oocytes [133].

### 2.11. PDE11

**Figure 11:**
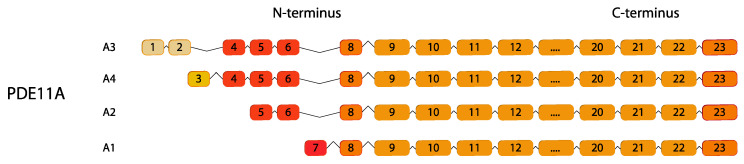
*PDE11* gene family. Human variants are depicted. This figure was gathered through analysis and cross-referencing of online databases (https://www.ensembl.org/ and https://www.uniprot.org, accessed on 30 March 2023). Boxes and lines represent exons and introns, respectively. The boxes with different colors indicate alternative exons. The maximum number of exons illustrated is 17 and ellipsis indicate exons not shown (adapted from [136]).

PDE11 is the most recently identified PDE and exhibits a dual substrate specificity for both cAMP and cGMP [134]. It is encoded by only one gene, *PDE11A*, that produces four variants (*PDE11A1*–*PDE11A4*) displaying different amino termini [135] (Figure 11).

In humans, PDE11A is relatively highly expressed in skeletal muscle and prostate while moderate expression levels have been detected in the testis, pituitary and thyroid glands [137]. Data on rodents suggest a role of PDE11 in sperm development and function since PDE11 is expressed at high levels in the testes and developing spermatozoa, and ejaculated sperm from *PDE11* knock-out mice showed lower spermatozoa counts and lower sperm motility reflecting a compromised fertilizing capacity [31,57] (see Table 1). Human spermatogonia, spermatocytes and spermatids as well as Leydig cells all express PDE11 [69] (Table 2). Data on human also come from a microarray analysis of PDEs expression showing that PDE11A is present at moderate levels in normal testis [64]. Even if presence of PDE11 in the testis is unquestionable, its effect on human sperm function is still unclear.

## 3. An Overview of Phosphodiesterase Families in Testicular Tumors

### 3.1. Testicular Tumors

Testicular tumors are the most common solid neoplasm of young adult men between 20–40 years of age. The two principal categories of testicular cancer comprise Testicular Germ Cells Tumors (TGCTs), which represent the majority of testis malignancies and “non-germ cell tumors”. Gonads are the elective site where these tumors arise; however, when the location is in extragonadal sites, they are called Extragonadal Germ Cell Tumor [138,139]. TGCTs can be distinguished according to their histological composition (Hematoxylin and Eosin and immunohistochemistry staining using specific markers), the germ cell lineage (aberrant development of the physiological germ cell at different phases of maturation) and the age of onset (pediatric, adolescent or adult).

TGCTs can arise from the precursor lesion called germ cell neoplasia in situ (GCNIS) that originates in fetal life. GCNIS remains dormant until puberty then, under hormonal influences, it starts to proliferate and initiate te invasive growth. Histologically, GCNIS transforms into seminoma or pure or mixed non-seminoma that includes embryonal cell carcinoma, choriocarcinoma, yolk sac tumors and teratomas. Seminomas, also referred to as Type II TGCTs, occur in adolescents and young adults (15 and 40 years of age), are the most common form of TGCTs and are always malignant. Type I and type III TGCTs do not arise from GCNIS and they occur in pediatric or in elderly men, respectively. Type I TGCTs are histologically subdivided into teratomatous tumors (benign) and yolk sac tumors (malignant). Type III TGCTs (spermatocytic seminoma, previously known as spermatocytic seminoma) contain cells that are similar to secondary spermatocytes [140].

Non-germ cells testicular tumors include a fair variety of neoplastic diseases. Among them, Leydig cell tumors (LCTs) represent the most common non-germ cell testicular tumors accounting for 3–22% of all testicular neoplasms [141,142,143,144]. Given the growing use of testis ultrasonography, but also increased exposure to endocrine disruptors [145,146], a progressive rise in the diagnosis of LCTs has been observed [145,146]. It is widely accepted that LCTs are always benign in the pediatric population whereas the malignant potential increases with age, peaking around 60 years of age. Sertoli tumors, the other type of non-germinal testicular tumors, are extremely rare accounting for only 1% of all testicular tumors.

### 3.2. Phosphodiesterases in Testicular Cancer

Neoplastic transformation can be driven by genetic and epigenetic changes, that in turn alter signaling pathways involved in the proper control of cell division, death and motility. cAMP and cGMP signaling participate in cell proliferation, energy homeostasis and metabolism [147,148,149,150,151] and when these signals become aberrant, we can assist in the onset of several pathological processes, including tumorigenesis [152,153]. The alteration of cAMP/cGMP by ACs/guanylyl cyclases, respectively, have been associated with both cyclic nucleotide synthesis or degradation by PDEs [154,155,156,157,158].

To identify gene signatures that may drive the development of seminoma, a gene expression profile was performed in seminoma samples and compared to normal testis. Chen and collaborators identified 1563 upregulated genes and 1939 downregulated genes. Among the downregulated pathways they found several metabolic signals, such as FoxO and Wnt, but more interestingly the cGMP-PKG signaling pathway [159]. Another gene that was found to be significantly associated with testicular cancer was PDE1A [160] and in vivo exposure of mice to secondhand smoke produced a unique ‘frameshift’ variant within the murine *PDE1A* suggesting an involvement of this PDE in non-familial testicular cancer [161].

**Figure 12:**
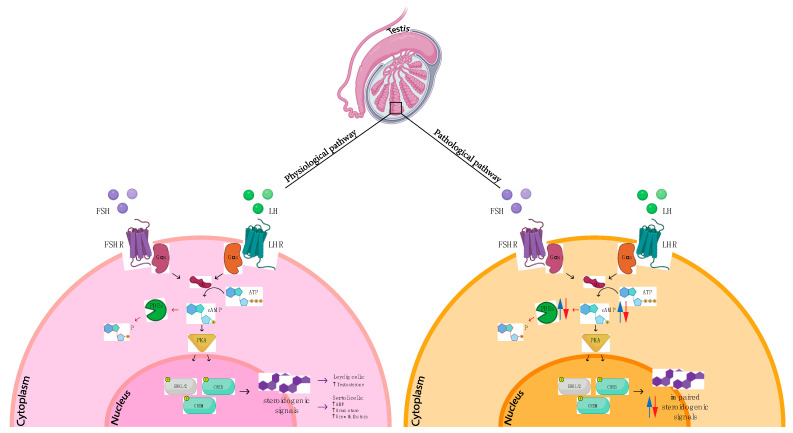
cAMP/PKA/PDEs mediated physiopathological processes in seminiferous tubules. LH: luteinizing hormone; FSH: follicle-stimulating hormone; LHR: luteinizing hormone receptor; FSHR: follicle-stimulating hormone receptor; Gs: G alpha subunit s protein; AC: adenylate cyclase; ATP: adenosine triphosphate; AMP: adenosine monophosphate; cAMP: cyclic adenosine monophosphate; PDEs: phosphodiesterases; PKA: protein kinase A; ERK1/2: extracellular signal-regulated kinase; CREB: cAMP-responsive element binding protein; CREM: cAMP-responsive element modulator; ABP: androgen binding protein.

PDE11A has been identified as another genetic modifying factor for the development of testicular tumors and it has been reported that PDE11A-inactivating variants may increase the risk of developing familial and bilateral testicular germ cell tumor. The first demonstration of this relationship comes from the observations of Horvart et al. who, sequencing *PDE11A* in 95 patients with TGCTs, identified several functional variants previously implicated in adrenal tumor predisposition [162]. This topic was further addressed by Azevedo et al., which reported inactivating germline mutations of *PDE11A* as modifiers of familial testicular germ cell tumors risk. After identifying PDE11 mutations, they transfected NTERA-2 and Tcam-2 cells with several mutated variants of *PDE11A* (R52T, F258Y, Y727C, R804H, V820M, R867G and M878V). They were able to demonstrate that cAMP levels were significantly higher, and the relative phosphodiesterase activity was lower in PDE11 mutated cells compared to wild-type cells [163]. A decisive contribution to this issue was given by the studies led by Pathak et al., indeed in a prior candidate gene study of 94 familial testicular germ cell tumors subjects, they were able to identify a significant correlation between the presence of functionally abnormal variants in *PDE11A* and a high risk to develop familial TGCT. They proposed a subsequent broader validation study sequencing the *PDE11A* coding region in 259 additional TGCT patients (both familial and sporadic) and 363 healthy controls. This analysis revealed the presence of more than 50 *PDE11A* variants, two of which were functionally characterized and shown to be functionally inactivating, resulting in reduced PDE activity and increased cAMP levels (Figure 12) [164]. Recently, Faja et al. analyzed PDE11 mutational status in semen from patients with unilateral and bilateral sporadic TGCTs and healthy controls. They were able to detect ten polymorphisms, not previously associated with testicular cancer, that are positively associated with TGCTs and correlating with sperm count [165].

PDEs misregulation has been reported also in non-germ testicular tumours. A higher frequency of *PDE11A* sequence variants in patients with large-cell calcifying Sertoli cell tumors was identified, pointing out how PDE11A could be considered a genetic modifying factor for the development of testicular tumors, acting directly on germ cells or indirectly through somatic cells [166]. Recently, we have demonstrated that during Leydig cell neoplastic transformation PDE8B expression levels increased compared to the normal testis, while PDE8A levels were almost comparable between the two sample groups suggesting, for the first time, a potentially pivotal role of PDE8B in LCs dysfunction [65] (Figure 12).

## 4. Discussion

In view of the enormous information available on PDEs in all tissues, it surprises that there is a remarkable paucity of studies regarding the presence, specific function and subcellular location of PDE subtypes in human testis and even less information on human testicular cancer is available.

Testis is a complex endocrine organ regulated by intra- and extra-testicular pathways that synergistically interact. In particular, mammalian spermatogenesis involves the interplay of different cell types and comprises a series of cellular and biochemical metamorphoses and the impairment of each step of this complex network could lead to neoplastic transformation. The single-cell types involved have been shown to express diverse PDEs and their localization and compartmentalization contribute to a spatiotemporal regulation of cAMP or cGMP [167].

cAMP-dependent signaling pathway, and to a lesser extent, cGMP signaling, are the main molecular mechanisms that played a major role in orchestrating the expression of the many genes in spermatogenesis [168,169,170,171].

Leydig cells have a crucial role in the regulation of steroidogenesis and spermatogenesis, since they are the production site of testosterone, which has a main role in fetal development and maturation, while the growth and differentiation of germ cells (i.e., the precursors of sperm) require Sertoli cells [172,173]. The synthesis and release of steroid hormones from Leydig cells, and the maturation of Sertoli cells, happen in response to two pituitary gonadotropins, LH and FSH. LH and FSH bind to the LH receptors and FSH receptors on Leydig and Sertoli cells, respectively, stimulating AC activity that raises intracellular cAMP level and activates PKA which regulates the expression of genes related to the steroidogenesis [174]. Substantial evidence for the regulatory function of PDEs in Leydig cells has been reported as a stimulatory effect of a pan-PDEi on testosterone release by primary LCs [175], indicating that one or more PDEs might be active in Leydig cells to modulate the intensity, duration and the desensitization of the LH-stimulated hormonal response [175] (Figure 12). Indeed, it was demonstrated that Leydig cells express transcripts for several cAMP-specific PDEs (Table 1 and Table 2) most of them contributing to Leydig cell response through LH receptor-cAMP signaling [52,176]. Testosterone is able to increase *PDE5A, PDE6D* and *PDE9A* expression [176]. Increased levels of cGMP in Leydig cells isolated from testosterone-treated rats confirmed an active role of cGMP-specific PDEs in degrading nitric oxide-stimulated cGMP [49].

Within the tubules, cGMP and cAMP cooperate in controlling germ cell differentiation, both directly and indirectly through Sertoli cells. cAMP-dependent signal transduction pathway, in particular, is one of the major regulatory mechanisms that operates at different stages of spermatogenesis. The regulation of gene expression is exerted via a family of nuclear transcription factors that bind to a specific DNA element designated cAMP-response element (CRE). The two predominant members of this family are the cAMP-response element binding protein (CREB) and the cAMP-responsive element modulator (CREM), which, in turn, transactivate the transcriptional expression of cAMP-responsive target genes [177,178]. The importance of CREM to male fertility was evident through the study of CREM knockout male mice, which are sterile having absolutely no mature spermatozoa [179], whereas CREB-mediated survival factor/s produced by Sertoli cells protect germ cell survival [180], strongly arguing for the essential role of CREB/CREM in sperm development in humans. Sperm function during capacitation, such as activation of motility, changes in the motility pattern known as hyperactivation have been attributed to the modulation of cAMP/cGMP [181].

Since 2017, mammalian testis transcriptomes, at different developmental stages and in both physiological and pathological conditions, have been extensively studied at the single-cell level by using single-cell RNA-sequencing (RNA-seq) revealing cell transcriptomes heterogeneities at a high resolution [182,183,184,185,186,187,188,189,190,191,192,193,194]. PDE transcripts do not appear as the top expressed gene or the most differentially expressed gene. Even though RNA-seq is a powerful tool it possesses some pitfall, it induces, for example, great RNA loss and low sequencing depth making it difficult to capture low abundant RNAs [195], thus causing an information loss. More information that is lost during RNAseq regards RNA isoform variants. Given that the testis is one of the organs that mostly exploit the potential of alternative splicing (AS) [196] and that, as mentioned, PDEs undergo extensive AS, it is conceivable that their transcripts have been underestimated. Moreover, it should be taken into account that, in germ cells, a temporal gap between mRNA transcription and protein synthesis exists, in part due to the fact that RNA synthesis terminates long before the spermatids complete their differentiation; therefore, it is mandatory to verify presence, specific function and subcellular location of the translated PDEs. Finally, a similar but not totally overlapping PDE expression pattern in testis between human and mouse tissues has been revealed. Taking this in mind, we demonstrated that in human testis, PDE8A and PDE8B localized in the cytosol in granular structure in Leydig cells as in mouse testis but more interestingly that PDE8A is expressed in round spermatids close to acrosome, and that it associates with the trans-Golgian region in specific stages, suggesting that it supports and sustains the trafficking of the vesicles originating from the Golgi apparatus for the acrosome biogenesis. We are encouraging a more systematic analysis of PDEs role/localization in the human testis to exploit unexpected functions of these enzymes.

Interference of cAMP/cGMP signaling pathway has been shown to be linked to tumorigenesis [155,197] and PDEs overexpression has been already described in several cancer types such happened for PDE11A and PDE8B, in adrenal hyperplasia/adenomas [162,198,199]. Indeed some PDEs have been already proposed as a potential biomarker for different tumoral contests [200,201,202,203,204] and we have proposed PDE8B as a promising biomarker for Leydig cell tumours. We believe that multi-omics will be useful to deeply analyze PDEs expression and activity for the translation of such findings in clinical practice. Moreover, PDE-opathies name has been coined to identify a set of disorders caused by germline mutations of PDEs [205], it would be interesting to extend this concept to testicular cancers with a more focused screening since their pharmacological inhibition has been proposed as an anticancer strategy in several tumors [18], but we are still far from the “they lived happily ever after”.

## Data Availability

Not applicable.
